# Characterization of rickettsiae in ticks in northeastern China

**DOI:** 10.1186/s13071-016-1764-2

**Published:** 2016-09-13

**Authors:** Huanhuan Liu, Qihong Li, Xiaozhuo Zhang, Zhongyu Li, Zedong Wang, Mingxin Song, Feng Wei, Shuchao Wang, Quan Liu

**Affiliations:** 1 College of Life Science, Jilin Agricultural University, Changchun, People’s Republic of China; 2 Military Veterinary Institute, Academy of Military Medical Sciences, Key Laboratory of Jilin Province for Zoonosis Prevention and Control, Changchun, People’s Republic of China; 3 Affiliated hospital of Academy of Military Medical Science, Beijing, People’s Republic of China; 4 College of Veterinary Medicine, Northeast Agricultural University, Harbin, People’s Republic of China

**Keywords:** Tick, *Rickettsia*, Rickettsiosis, Northeastern China

## Abstract

**Background:**

Tick-borne rickettsioses are considered important emerging zoonoses worldwide, but their etiological agents, rickettsiae, remain poorly characterized in northeastern China, where many human cases have been reported during the past several years. Here, we determined the characteristics of *Rickettsia* spp. infections in ticks in this area.

**Methods:**

Ticks were collected by flagging vegetation from Jilin and Heilongjiang provinces of northeastern China followed by morphological identification. The presence of *Rickettsia* spp. in ticks was detected by PCR targeting the 23S-5S ribosomal RNA intergenic spacer, citrate synthase (*gltA*) gene, and 190-kDa outer membrane protein gene (*ompA*). The newly-generated sequences were subjected to phylogenetic analysis using the software MEGA 6.0.

**Results:**

The overall infection rate of *Rickettsia* spp. was 6.12 %. Phylogenetic analyses based on the partial *gltA* and *ompA* genes demonstrated that rickettsiae detected in the ticks belong to four species, including “*Candidatus* Rickettsia tarasevichiae”, *Rickettsia heilongjiangensis*, *Rickettsia raoultii*, and a potential new species isolate. The associated tick species were also identified, i.e. *Dermacentor nuttalli* and *Dermacentor silvarum* for *R. raoultii*, *Haemaphysalis concinna* and *Haemaphysalis longicornis* for *R. heilongjiangensis*, and *Ixodes persulcatus* for “*Ca.* R. tarasevichiae”. All *Rickettsia* spp. showed significantly high infection rates in ticks from Heilongjiang when compared to Jilin Province.

**Conclusion:**

*Rickettsia heilongjiangensis*, *R. raoultii* and “*Ca.* R. tarasevichiae” are widely present in the associated ticks in northeastern China, but more prevalent in Heilongjiang Province. The data of this study increase the information on the distribution of *Rickettsia* spp. in northeastern China, which have important public health implications in consideration of their recent association with human diseases.

**Electronic supplementary material:**

The online version of this article (doi:10.1186/s13071-016-1764-2) contains supplementary material, which is available to authorized users.

## Background

Tick-transmitted diseases have become an increasing public health problem [1–3]. Tick-borne rickettsioses are considered important emerging zoonoses worldwide, due to tick distribution alterations, shifting climates, and accelerating urbanization [4, 5]. These diseases share characteristic clinical features, including fever, asthenia, anorexia, nausea, headache, rash and occasional eschar formation at the site of the tick bite.

At least nine species or subspecies of tick-borne rickettsiae have been identified during the past 30 years in China, including *Rickettsia heilongjiangensis*, *Rickettsia sibirica* sp BJ-90, *Rickettsia raoultii* and “*Candidatus* Rickettsia tarasevichiae” [6–9]. *Rickettsia heilongjiangensis* was primarily identified in Suifenhe and Luobei of Heilongjiang Province in 1984, and isolated from the blood of a tick-bitten patient in the same place ten years later [10–12]. Since then, this organism has been detected or isolated in other countries, including Russia, Japan and Thailand [13–15]. *Rickettsia heilongjangensis* was first proven responsible for human disease in 1996, and 34 human cases have been reported [3]. *Rickettsia sibirica* strain BJ-90 was primarily detected in *Dermacentor sinicus* ticks from Beijing, and had not been considered as a pathogenic agent for humans until 2012 when an old farmer from Mudanjiang of Heilongjiang Province was diagnosed with infection of this organism [7]. *Rickettsia raoultii*, initially detected in Russia in 1999 and considered a novel species in 2008, is prevalent in various regions of Asia and Europe [16–19]. In 2009, *R. raoultii* was determined as a human pathogen in France [20]. In China, DNA of this bacterium was first detected in Jilin Province in 2008 [21]. Recently, *R. raoultii* has been found prevalent in Heilongjiang, Xinjiang and Tibet provinces [22–25]. However, the first human case of *R. raoultii* infection in China was reported in the northeast in 2014 [9]. “*Ca* R. tarasevichiae”, belonging to the so-called ancestral group that was traditionally considered nonpathogenic, was first detected in *Ixodes persulcatus* ticks collected from various regions of Russia, and subsequently recorded in a wide territory from Estonia to Japan [26–28]. In 2013, specific DNA of “*Ca.* R. tarasevichiae” was detected in blood samples of five misdiagnosed patients in northeastern China [8].

More than 500 human cases have been reported in China in the past 13 years, mostly in the northeastern region of the country, suggesting an increasing risk of *Rickettsia* infection. We therefore conducted this study to detect and characterize the rickettsiae in ticks collected in northeastern China.

## Methods

### Collection of ticks

**Fig. 1:**
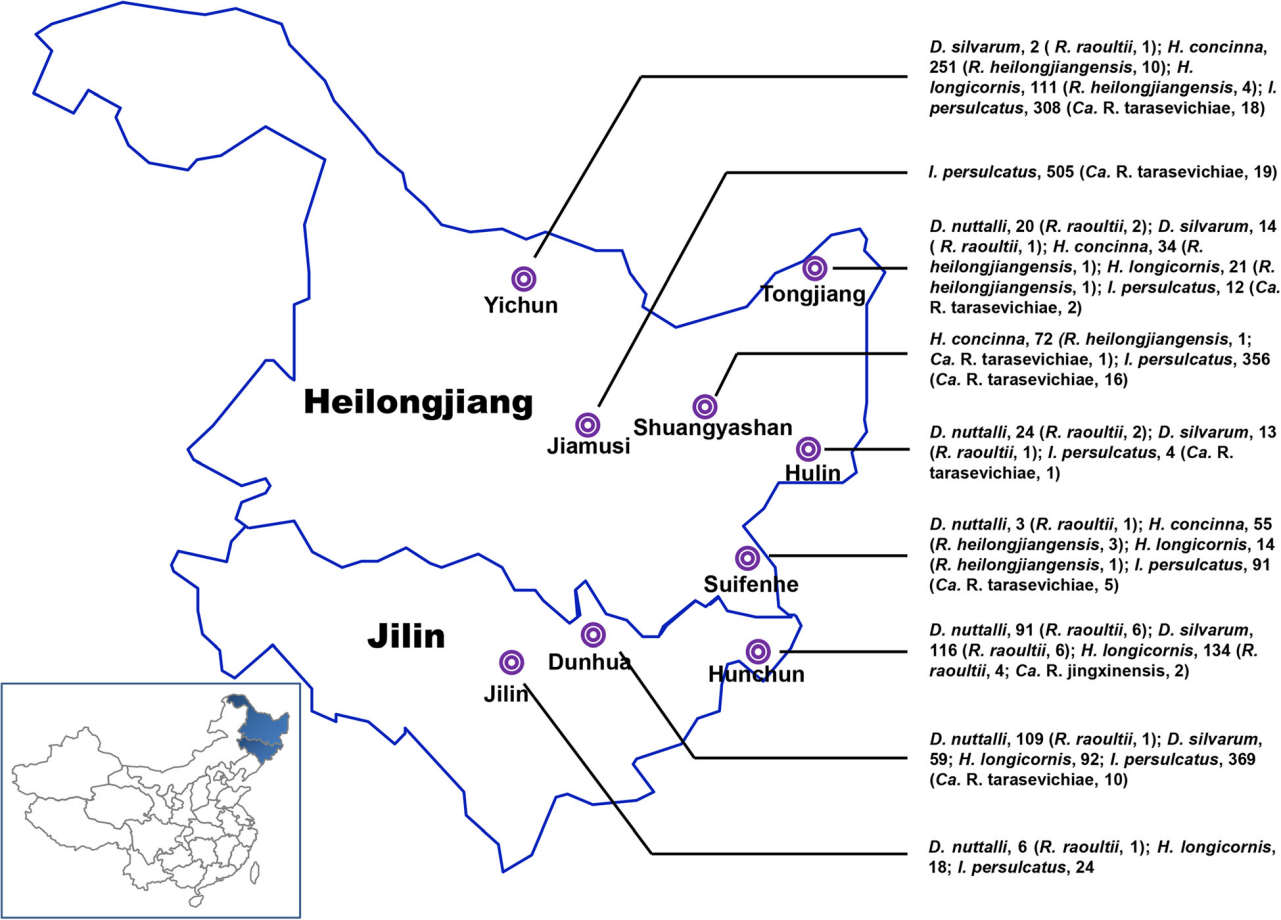
Sampling sites, ticks and rickettsiae. Ticks were collected from various regions in Jilin and Heilongjiang provinces. Tick species and the rickettsiae detected are shown in parentheses

During April and May 2015, ticks were collected by flagging vegetation in nine regions of northeastern China, including Jilin (42°39′–43°20′N, 126°12′–127°23′E), Dunhua (42°27′–43°2′N, 129°50′–130°44′E) and Hunchun (42°47′–43°6′N, 130°6′–130°12′E) located in Jilin Province, and Yichun (47°24′–48°3′N, 128°23′–129°37′E), Jiamusi (46°43′–47°11′N, 133°12′–133°56′E), Shuangyashan (46°27′–46°58′N, 131°12′–131°23′E), Tongjiang (47°43′–48°3′N, 133°29′–133°44′E), Hulin (45°33′–45°54′N, 132°49′–133°23′E) and Suifenhe (44°23′N, 131°9′E) situated in Heilongjiang Province (Fig. 1). The tick species were identified following morphological criteria as described previously or using molecular biology tools after PCR targeting the 16S ribosomal RNA gene with the forward primer TickHF (5′-GGT ATT TTG ACT ATA CAA AGG TAT TG-3′) and the reverse primer TickHR (5′-TTA TTA CGC TGT TAT CCC TAG AGT ATT-3′) [29].

### Detection of rickettsiae

The sampled ticks were pooled, approximately 15 ticks per pool, based on the tick species and sampling sites. After washing with 70 % ethanol and double distilled water, the pooled ticks were homogenized in 1 ml sterile PBS. DNA was extracted from 200 μl tick homogenates using TIANamp Genomic DNA Purification System (TIANGEN, Beijing, China) according to the manufacturer’s instructions. The tick pools were initially screened for the presence of *Rickettsia* spp. by amplifying the 23S-5S rRNA intergenic spacer by polymerase chain reaction (PCR) using the primers RCK/23-5-F and RCK/23-5-R as described previously [30]. Double-distilled water and a previously determined positive sample were used as negative and positive controls, respectively.

The positive pools were subsequently analyzed by amplifying the partial citrate synthase (*gltA*) gene and outer membrane protein A (*ompA*) gene of *Rickettsia* spp. by PCR with the primers CS2d and CSEndr for *gltA* targeting a 1289 bp fragment, and Rr190.70p and Rr190.602n for *ompA* targeting a 533 bp fragment [8, 31]. The PCR products were cloned into pMD18-T vector (Takara, Dalian, China) and sequenced.

### Phylogenetic analysis

The newly-generated sequences were aligned exclusively, or together with those retrieved from the GenBank database using the software ClustalW 2.0. Phylogenetic trees were generated in a Maximum Likelihood analysis using the software Molecular Evolutionary Genetics Analysis (MEGA) version 6.0 [11].

### Statistical analysis

The infection rates of *Rickettsia* spp. in ticks were calculated using the software PooledInfRate version 4.0 (By Biggerstaff, Brad J., a Microsoft® Office Excel^©^ Add-In to compute prevalence estimates from pooled samples. Centers for Disease Control and Prevention, Fort Collins, CO, USA, 2009). The software provides three computing methods for infection rate estimation, including bias-corrected maximum likelihood estimation (Bias-corrected MLE) and minimal infection rate (MIR). The former exhibits better accuracy than the latter, but requires that not all the pools are positive. Statistical significance was evaluated by Fisher’s exact test and Chi-square test; *P* < 0.05 is considered significant.

## Results

A total of 2928 ticks, including 2813 adults and 115 nymphs, were collected from Jilin and Heilongjiang provinces of northeastern China (Fig. 1). In particular, the nymphs were exclusively collected from Jingxin town (42°30′N, 130°38′E) in Jilin Province. The species of ticks were morphologically determined as *Dermacentor nuttalli* (*n* = 253), *Dermacentor silvarum* (*n* = 204), *Haemaphysalis concinna* (*n* = 412), *Haemaphysalis longicornis* (*n* = 390, 275 adults and 115 nymphs) and *Ixodes persulcatus* (*n* = 1669). Based on species and sampling site, the identified ticks were subsequently assigned into 204 pools, of which 201 were adults and three were nymphs (detailed in Additional file 1: Table S1). In order to ensure the accuracy of identification of tick species, we also analyzed the partial sequence of 16S ribosomal RNA gene of a portion of ticks using BLAST. The sequences (354 nt, GenBank accession no. KX305956) derived from five tick pools which were morphologically determined as *I. persulcatus* were genetically identical to one another and presented 100 % similarity to that of *I. persulcatus* isolate Irk5m (accession no. JF934741.1). The partial 16S rRNA gene sequences (252 nt, accession no. KX305957) obtained from *D. nuttalli* pools were also identical to each other but differed at two nucleotide positions from that of the nearest tick species (*D. nuttalli* isolate XJ088, accession no. JX051114.1). The three pools of nymphs and the adult *H. longicormis* ticks shared the same partial nucleotide sequence (127 nt, accession no. KX305958) of the 16S rRNA gene that was most related (99 % similarity) to the sequence of *H. longicormis* isolate YN07 (accession no. JX051064.1). With the exception of *H. concinna* absent in samples from Jilin, all these tick species were found in both provinces. *Dermacentor* species were more prevalent (Pearson Chi-square test; *χ*^2^ = 564.0, *df *= 1, *P* < 0.0001) in Jilin (37.4 %) in comparison with Heilongjiang (4.0 %), where *I. persulcatus* was the predominant tick species (Table 1).

**Table 1:** PCR survey results for ticks tested for rickettsiae, northeastern China, 2015. Infection rates of *Rickettsia* spp. in ticks were calculated following the Bias-corrected MLE method in the software Pooledinfrate, version 4.0; 95 % confidence intervals (CI) are presented in brackets

Tick species	No. of ticks tested	Total no. (%) ticks positive [95 % CI]	Jilin					Heilongjiang				
			Total no. of ticks tested	Total no. (%) ticks positive [95 % CI]	*R. r.* (%) [95 % CI]	*C. R. j.* (%) [95 % CI]	*C. R. t.* (%) [95 % CI]	Total no. of ticks tested	Total no. of (%) ticks positive	*R. r.* (%) [95 % CI]	*R. h.* (%) [95 % CI]	*C. R. t.* (%) [95 % CI]
*Dermacentor nuttalli*	253	13 (7.45) [4.30–12.59]	206	8 (5.16) [2.48–10.02]	8 (5.16) [2.48–10.02]	0	0	47	5 (10.64)^a^	5 (10.64)^a^	0	0
*Dermacentor silvarum*	204	9 (6.07) [3.10–11.37]	175	6 (4.42) [1.86–9.44]	6 (4.42) [1.86–9.44]	0	0	29	3 (10.30)^a^	3 (10.30)^a^	0	0
*Haemaphysalis concinna*	412	16 (5.46) [3.28–8.80]	0	0	0	0	0	412	16 (5.46) [3.28–8.80]	0	15 (4.96) [2.93–8.09]	1 (0.24) [0.01–1.18]
*Haemaphysalis longicornis*	390	12 (3.91) [2.23–6.59]	244	6 (2.89) [1.30–5.99]	4 (1.58) [0.58–3.61]	2 (0.92) [0.16–3.2]	0	146	6 (5.42) [2.29–11.64]	0	6 (5.42) [2.29–11.64]	0
*Ixodes persulcatus*	1,669	72 (6.53) [5.16–8.22]	393	10 (3.12) [1.61–5.62]	0	0	10 (3.12) [1.61–5.62]	1,276	62 (7.95) [6.16–10.22]	0	0	62 (7.95) [6.16–10.22]
Total	2,928	122 (6.12) [5.13–7.29]	1,018	30 (3.79) [2.64–5.34]	18 (1.95) [1.22–2.99]	2 (0.20) [0.04–0.67]	10 (1.05) [0.54–1.87]	1,910	92 (7.66) [6.23–9.39]	8 (0.42) [0.20–0.80]	21 (1.16) [0.76–1.78]	63 (4.36) [3.39–5.54]

*Abbreviations*: *R. r.*, *Rickettsia raoultii*; *C. R. j.*, “*Candidatus* Rickettsia jingxinensis”; *C. R. t.*, “*Candidatus* Rickettsia tarasevichiae”; *R. h*., *Rickettsia heilongjiangensis*

^a^Infection rates are calculated using the MIR method

**Fig. 2:**
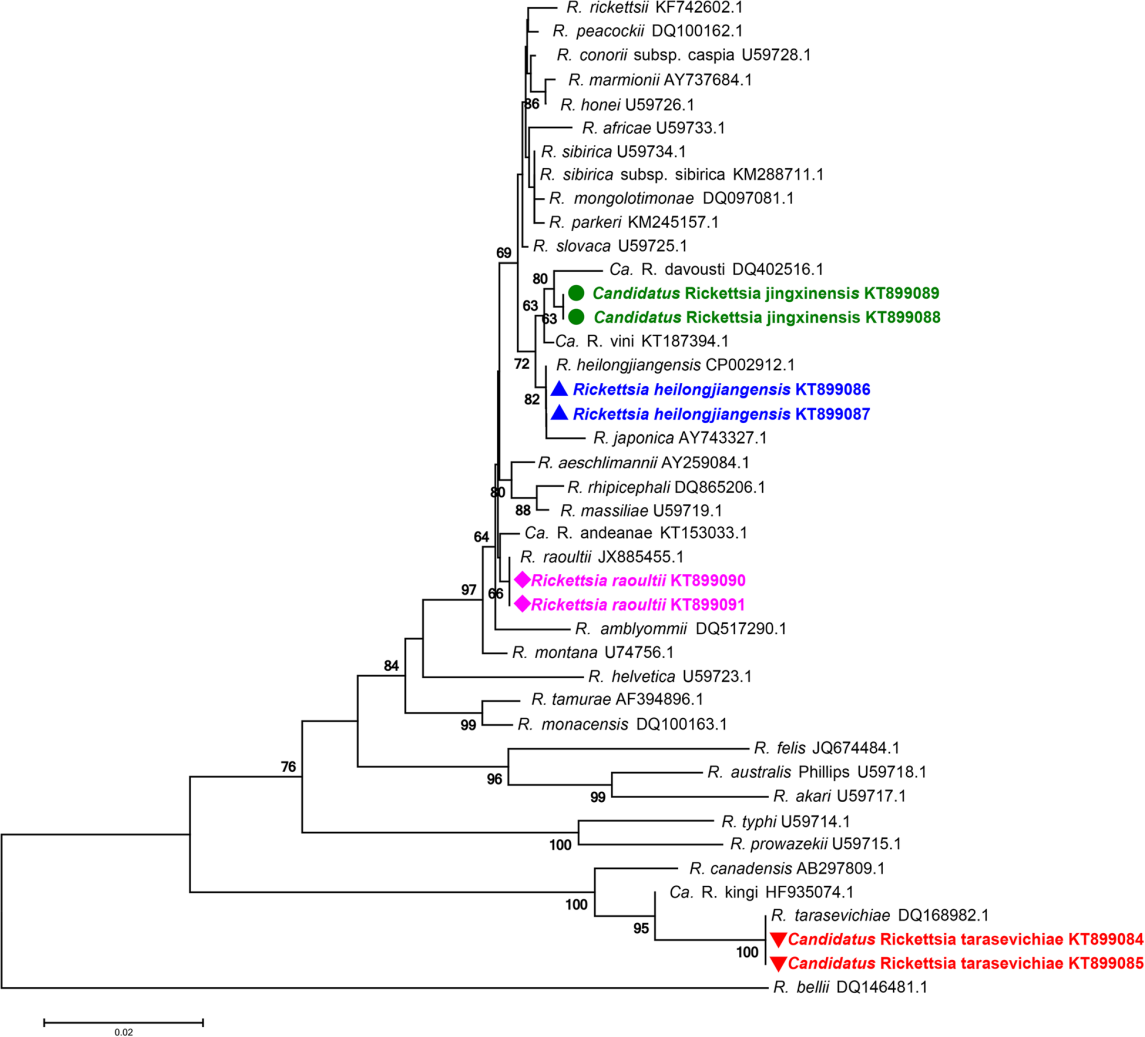
Phylogenetic trees based on partial sequences of *gltA* (1289 bp) gene. Sequences of the *Rickettsia* species detected in the present study were aligned with those retrieved from the GenBank database. Phylogenetic analysis was performed using the Maximum Likelihood method and trees were tested by bootstrapping (1000 pseudoreplicates). *Rickettsia bellii* was used as the outgroup. The scale-bar indicates the number of substitutions (Kimura 2-parameter model) per site. The scale-bar indicates the number of substitutions (Kimura 2-parameter model) per site. Sequences for species detected in the present study are indicated by geometric shapes and colours

**Fig. 3:**
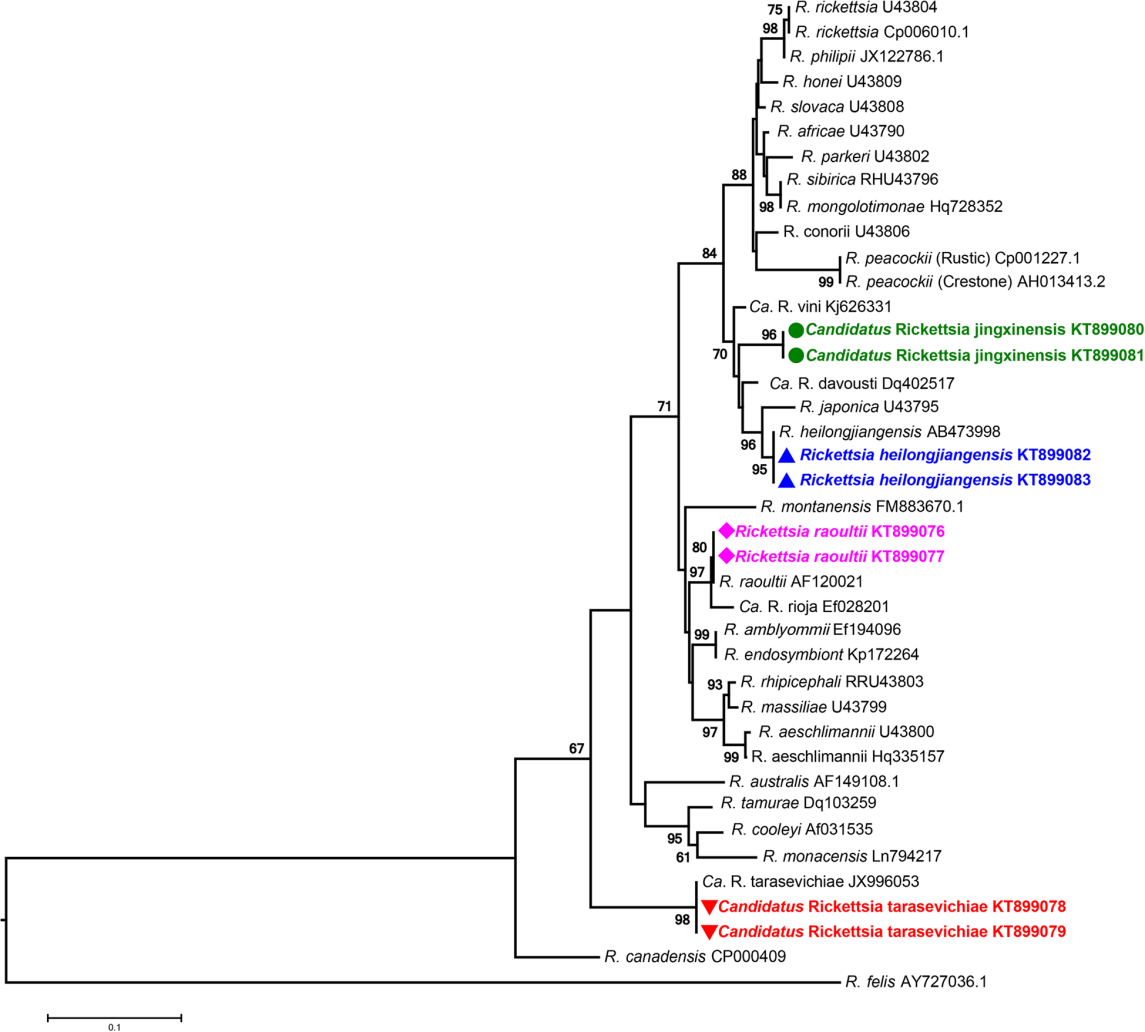
Phylogenetic tree based on partial sequences of *ompA* (533 bp) gene. Sequences of the *Rickettsia* species detected in the present study were aligned with those retrieved from the GenBank database. Phylogenetic analysis was performed using the Maximum Likelihood method and trees were tested by bootstrapping (1000 pseudoreplicates); *Rickettsia felis* was used as the outgroup. The scale-bar indicates the number of substitutions (Kimura 2-parameter model) per site. Sequences for species detected in the present study are indicated by geometric shapes and colours

To investigate the presence of *Rickettsia* spp. in tick samples, molecular screening was first performed using the universal primer set. In total, 122 tick pools out of 2928 (6.12 %) ticks were found positive for *Rickettsia* spp. To identify the *Rickettsia* spp. in these positive samples, partial *gltA* (1289 bp) and *ompA* (533 bp) gene sequences were further obtained and sequenced. Phylogenetic analysis revealed that these sequences could be clustered into four clades (Additional file 2: Figure S1). After BLAST on the NCBI website, sequences in the GenBank database most similar to the query sequences were retrieved and used for phylogenetic analysis, revealing that clades 1, 2 and 4 were corresponding to “*Ca.* R. tarasevichiae”, *R. heilongjiangensis* and *R. raoultii*, respectively (Fig. 2 and Fig. 3). Two identical sequences obtained from *H. longicornis* collected in Jingxin town of Hunchun city constituted the clade 3. The affirmatory species of *Rickettsia* most-related to clade 3 was “*Candidatus* Rickettsia vini” which shared 96.6 % nucleotide similarity of *ompA* sequence and 99.7 % of *gltA* sequence. Current criteria for sequence-based classification of a *Rickettsia* species as a new “*Candidatus* Rickettsia” species requires the sequence similarity of the newly identified species with the established *Rickettsia* spp. should be: < 99.9 % for *gltA* and < 98.8 % for *ompA* [11]. Phylogenetic analyses based on *gltA* and *ompA* sequence showed the clade 3 as an independent clade (Fig. 2 and Fig. 3). These results suggest that species in the clade 3 could be a potential new *Rickettsia* species. We propose to provisionally designate it as “*Candidatus* Rickettsia jingxinensis”.

The overall infection rate of rickattsiae in *Dermacentor* ticks in Jilin and Heilongjiang provinces (4.42–5.16 % and 10.30–10.64 %, respectively) were higher than those of the other tick species (0–3.12 % and 5.42–7.95 %, respectively). The infection rate of rickettsiae in ticks from Heilongjiang were significantly higher than that of Jilin (Pearson Chi-square test; *χ*^2^ = 5.355, *df* = 1, *P* = 0.0207) (Table 1).

*Rickettsia raoultii* was detected with comparable infection rate in both *D. nuttalli* and *D. silvarum* with infection rate strikingly higher in Heilongjiang (10.30–10.64 %) as compared to Jilin (4.42–5.16 %) (Fisher’s exact test; *χ*^2^ = 6.595, *df* = 1, *P* = 0.017). *Haemaphysalis longicormis* from Jilin Province was also detected positive (1.58 %) for *R. raoultii* (Table 1). The potential new *Rickettsia* species “*Ca.* R. jingxinensis” was merely found in two tick pools of nymphs of *H. longicormis* from Jingxin in Jilin Province with an infection rate of 0.92 %. DNA of *R. heilongjiangensis* was exclusively detected in *Haemaphysalis* ticks from Heilongjiang Province with infection rate of 4.96 % in *H. concinna* and 5.42 % in *H. longicormis*. “*Ca.* R. tarasevichiae” DNA was only present in *I. persulcatus* with one exception in *H. longicormis* collected from Shuangyashan of Heilongjiang Province (Fig. 1).

The representative partial sequences of *gltA* and *ompA* gene in the present study have been deposited to Genbank (see accession numbers in Table 2).

**Table 2:** GenBank accession numbers of the sequences generated in the present study

Species	*gltA*	*ompA*
*R. raoultii* in *D. nuttalli*	KT899090	KT899076
*R. raoultii* in *H. longicornis*,	KT899091	KT899077
“*Candidatus* R. tarasevichiae” in *H. concinna*	KT899084	KT899078
“*Candidatus* R. tarasevichiae” in *I. persulcatus*	KT899085	KT899079
“*Candidatus* R. jingxinensis” in *H. longicornis*	KT899088	KT899080
*R. heilongjiangensis* in *H. concinna*	KT899086	KT899082
*R. heilongjiangensis* in *H. longicornis*	KT899087	KT899083

## Discussion

To date, twenty-one tick species of seven genera have been reported in northeastern China [32]. In the present study, we only collected five tick species, including *D. nuttalli*, *D. silvarum*, *H. concinna*, *H. longicornis* and *I. persulcatus*. We also demonstrated that the tick population in Jilin Province is different from that of Heilongjiang Province. For example, *Dermacentor* species are predominant in Jilin, compared with Heilongjiang Province. *Haemaphysalis concinna* had been reported in Hunchun and Dunhua (Jilin Province) [32], but it was not found in this study. The possible reasons may come from the limitation of sampling period and regions, and altered tick population induced by the interruption of nature balance [33].

All of the tick species identified in our study can function as vectors that could transmit various pathogens to humans and animals. For instance, *H. longicornis* is a vector for *R. heilongjiangensis*, “*Candidatus* Rickettsia hebeiii”, *Ehrilchia chaffeensis*, *Borrelia garinii* and *Babesia mircroti* [3]. In northeastern China, *Rickettsia* spp. spread by human-bitten ticks could be a serious public health problem, and several *Rickettsia* spp. have been identified or isolated from ticks or patients, including *R. heilongjiangensis*, *Rickettsia sibrica*, *Rickettsia japonica*, *R. raoultii* and “*Ca.* R. tarasevichiae” during the past 30 years [3]. In the current study, three *Rickettsia* spp., including *R. heilongjiangensis*, *R. raoultii*, “*Ca.* R. tarasevichiae”, and a potential new species “*Ca.* R. jingxinensis”, were detected in ticks, with an overall infection rate of 6.12 %. Previous reports showed that *Rickettsia* infection rates ranged from 1.53 to 32.25 % in a certain species, or a certain vector, or a certain origin of northeastern China [21, 34]. Intriguingly, the infection rates of rickettsiae in Heilongjiang Province were found to be strikingly higher than those of Jilin Province in the present study. The geography-based dissimilarity of *Rickettsia* presence provides new insight for the prevention and control of tick-borne rickettsioses. In northeastern China, *R. heilongjiangensis* was detected in three counties of Heilongjiang with an infection rate of 4.7 % [15]. *Rickettsia heilongjiangensis* was initially detected in *D. silvarum* and *Haemaphysalis* ticks [12], but only found in the latter in this study, which confirmed the previous finding that *Haemaphysalis* ticks were the major vector of *R. heilongjiangensis* [35]. Despite the presence of *R. heilongjiangensis* in Jilin Province was proven in rodent animals and humans [15], we only detected it in ticks from Heilongjiang Province in the current study. This result suggests *R. heilongjiangensis* could be more prevalent in Heilongjiang Province. At the China-Russia border, *R. raoultii* was considered to be the predominant *Rickettsia* in *D. silvarum*; this was confirmed in the present study [22]. Furthermore, we also demonstrated that in Jilin and Heilongjiang provinces, *R. raoultii* appeared to be predominant in *D. nuttalli*, as already shown in a study in Mongolia [36]. Although *R. raoultii* was detected in other tick species, such as *Haemaphysalis erinacei* and *I. persulcatus*, we did not amplify DNAs of this *Rickettsia* species from ticks except for *Dermacentor* species, suggesting that *Dermacentor* species may be the major vector for *R. raoultii*, as stated in the previous studies [22, 23]. “*Ca.* R. tarasevichiae” was recently found in patients and *I. persulcatus* in Heilongjiang Province in China, renewing the old concept that members of the ancestral group of *Rickettsia* were nonpathogenic [8, 34]. In this study, the presence of “*Ca.* R. tarasevichiae” was extended from Heilongjiang to Jilin Province. For the first time, we detected “*Ca.* R. tarasevichiae” in *H. longicornis* in China, which confirmed the presence of this organism in tick species besides *I. persulcatus*, as reported in Russia [37].

In addition, we also detected a new variant “*Ca.* R. jingxinensis”*.* Phylogenetic analyses based on both *ompA* and *gltA* gene sequences indicated this may be a new species. However, additional studies are required to verify this possibility. “*Ca.* R. jingxinensis” is closely related to “*Ca.* Rickettsia vini” and “*Ca. Rickettsia davousti*”. Although the *Candidatus* species have not been identified as human or animal pathogens despite a wide geographic distribution, we could not exclude the potential threat for humans and animals [38, 39].

Our study had some limitations. First, our investigation was subjected to bias because the infection rates were calculated in pooled samples, and we could not exclude the possibility of various strains in each pool. Therefore, the actual infection rates might be higher than stated above. Second, since our interests mainly focused on the infection rates in different areas and tick species in northeastern China, we did not identify the sex of the ticks. Thus, it is impossible to ascertain the precise infection rate of *Rickettsia* spp. based on the vector gender in this study.

## Conclusion

We determined that *D. nuttalli*, *D. silvarum*, *H. concinna*, *H. longicornis* and especially *I. persulcatus* were the major tick species, and acting mainly as vectors for *R. raoultii*, *R. heilongjiangensis* and “*Ca.* R. tarasevichiae”, respectively, in northeastern China. These rickettsiae were more prevalent in Heilongjiang as compared to Jilin Province. These data increase the information on the distribution of rickettsiae in northeastern China, which have important public health implications in consideration of their recent association with human diseases.

## Additional files

Additional file 1:
**Table S1.** Ticks and tick-borne *Rickettsia* spp. (DOCX 23 kb) Additional file 2:
**Figure S1.** Phylogenetic analysis of the partial *ompA* (533 bp) sequences obtained in this study. Targeted fragments of *ompA* gene of all of the positive samples were analyzed through the Maximum Likelihood method using MEGA 6.0 [40]. Phylogenic tree was tested by bootstrapping (1000 pseudoreplicates). Sequences in the tree are identified by sample number, tick species, and sampling site. (TIF 5444 kb)

## Abbreviations

rRNA: Ribosomal RNA
*gltA*: Citrate synthase gene
*ompA*: 190-kDa outer membrane protein gene
