# Supplementary material for: Characterization of rickettsiae in ticks in northeastern China
Source: Parasit Vectors. 2016 Sep 13;9(1):498. doi: 10.1186/s13071-016-1764-2 (PMC5022169; doi:10.1186/s13071-016-1764-2)
Supplement: Additional file 1: — Table S1. Ticks and tick-borne Rickettsia spp. (DOCX 23 kb) [file 13071_2016_1764_MOESM1_ESM.docx]

**Additional file 1: Table S1.** Ticks and tick-borne *Rickettsia* spp.

| **Pool ID** | **Tick species** | **Sampling region** | ***Rickettsia* species** | **Pool size** |
| --- | --- | --- | --- | --- |
| 1 | *Dermacentor nuttalli* | Jilin-Hunchun | *Rickettsia raoultii* | 16 |
| 2 | *Dermacentor nuttalli* | Jilin-Hunchun | *Rickettsia raoultii* | 15 |
| 3 | *Dermacentor nuttalli* | Jilin-Hunchun | *Rickettsia raoultii* | 15 |
| 4 | *Dermacentor nuttalli* | Jilin-Hunchun | *Rickettsia raoultii* | 15 |
| 5 | *Dermacentor nuttalli* | Jilin-Hunchun | *Rickettsia raoultii* | 15 |
| 6 | *Dermacentor nuttalli* | Jilin-Hunchun | *Rickettsia raoultii* | 15 |
| 14 | *Dermacentor silvarum* | Jilin-Hunchun | *Rickettsia raoultii* | 15 |
| 15 | *Dermacentor silvarum* | Jilin-Hunchun | *Rickettsia raoultii* | 15 |
| 16 | *Dermacentor silvarum* | Jilin-Hunchun | *Rickettsia raoultii* | 15 |
| 17 | *Dermacentor silvarum* | Jilin-Hunchun | *Rickettsia raoultii* | 15 |
| 18 | *Dermacentor silvarum* | Jilin-Hunchun | *Rickettsia raoultii* | 15 |
| 19 | *Dermacentor silvarum* | Jilin-Hunchun | *Rickettsia raoultii* | 14 |
| 29 | *Dermacentor silvarum* | Jilin-Hunchun | Negative | 18 |
| 30 | *Dermacentor silvarum* | Jilin-Hunchun | Negative | 9 |
| 31 | *Haemaphysalis longicornis* | Jilin-Hunchun | *Rickettsia raoultii* | 4 |
| 32 | *Haemaphysalis longicornis* | Jilin-Hunchun | *Rickettsia raoultii* | 4 |
| 33 | *Haemaphysalis longicornis* | Jilin-Hunchun | *Rickettsia raoultii* | 4 |
| 34 | *Haemaphysalis longicornis* | Jilin-Hunchun | *Rickettsia raoultii* | 4 |
| 35 | *Haemaphysalis longicornis* | Jilin-Hunchun | Negative | 3 |
| 46 | *Ixodes persulcatus* | Jilin-Dunhua | "*Candidatus* Rickettsia tarasevichiae" | 23 |
| 47 | *Haemaphysalis longicornis* | Jilin-Dunhua | Negative | 12 |
| 48 | *Haemaphysalis longicornis* (Nymphs) | Jilin-Hunchun | Negative | 39 |
| 49 | *Haemaphysalis longicornis* (Nymphs) | Jilin-Hunchun | *Candidatus Rickettsia jingxinensis.* | 38 |
| 50 | *Haemaphysalis longicornis* (Nymphs) | Jilin-Hunchun | *Candidatus Rickettsia jingxinensis.* | 38 |
| 51 | *Dermacentor silvarum* | Jilin-Dunhua | Negative | 15 |
| 52 | *Dermacentor silvarum* | Jilin-Dunhua | Negative | 15 |
| 53 | *Dermacentor silvarum* | Jilin-Dunhua | Negative | 15 |
| 54 | *Dermacentor silvarum* | Jilin-Dunhua | Negative | 14 |
| 55 | *Ixodes persulcatus* | Jilin-Dunhua | "*Candidatus* Rickettsia tarasevichiae" | 15 |
| 56 | *Ixodes persulcatus* | Jilin-Dunhua | "*Candidatus* Rickettsia tarasevichiae" | 15 |
| 57 | *Ixodes persulcatus* | Jilin-Dunhua | "*Candidatus* Rickettsia tarasevichiae" | 15 |
| 58 | *Ixodes persulcatus* | Jilin-Dunhua | "*Candidatus* Rickettsia tarasevichiae" | 15 |
| 59 | *Ixodes persulcatus* | Jilin-Dunhua | "*Candidatus* Rickettsia tarasevichiae" | 15 |
| 60 | *Ixodes persulcatus* | Jilin-Dunhua | "*Candidatus* Rickettsia tarasevichiae" | 15 |
| 61 | *Ixodes persulcatus* | Jilin-Dunhua | Negative | 14 |
| 62 | *Ixodes persulcatus* | Jilin-Dunhua | "*Candidatus* Rickettsia tarasevichiae" | 14 |
| 63 | *Ixodes persulcatus* | Jilin-Dunhua | Negative | 14 |
| 64 | *Ixodes persulcatus* | Jilin-Dunhua | Negative | 14 |
| 65 | *Ixodes persulcatus* | Jilin-Dunhua | Negative | 14 |
| 66 | *Dermacentor nuttalli* | Jilin-Dunhua | Negative | 14 |
| 67 | *Dermacentor nuttalli* | Jilin-Dunhua | Negative | 14 |
| 68 | *Dermacentor nuttalli* | Jilin-Dunhua | Negative | 14 |
| 69 | *Dermacentor nuttalli* | Jilin-Dunhua | Negative | 14 |
| 70 | *Dermacentor nuttalli* | Jilin-Dunhua | Negative | 14 |
| 71 | *Dermacentor nuttalli* | Jilin-Dunhua | Negative | 13 |
| 72 | *Dermacentor nuttalli* | Jilin-Dunhua | Negative | 13 |
| 73 | *Ixodes persulcatus* | Jilin-Dunhua | Negative | 15 |
| 74 | *Ixodes persulcatus* | Jilin-Dunhua | "*Candidatus* Rickettsia tarasevichiae" | 15 |
| 75 | *Ixodes persulcatus* | Jilin-Dunhua | Negative | 15 |
| 76 | *Ixodes persulcatus* | Jilin-Dunhua | Negative | 15 |
| 77 | *Ixodes persulcatus* | Jilin-Dunhua | Negative | 14 |
| 78 | *Ixodes persulcatus* | Jilin-Dunhua | Negative | 14 |
| 79 | *Ixodes persulcatus* | Jilin-Dunhua | Negative | 14 |
| 80 | *Ixodes persulcatus* | Jilin-Dunhua | Negative | 14 |
| 81 | *Ixodes persulcatus* | Jilin-Dunhua | "*Candidatus* Rickettsia tarasevichiae" | 14 |
| 82 | *Ixodes persulcatus* | Jilin-Dunhua | Negative | 14 |
| 83 | *Ixodes persulcatus* | Jilin-Dunhua | Negative | 14 |
| 84 | *Ixodes persulcatus* | Jilin-Dunhua | Negative | 14 |
| 85 | *Haemaphysalis longicornis* | Jilin-Dunhua | Negative | 13 |
| 86 | *Haemaphysalis longicornis* | Jilin-Dunhua | Negative | 13 |
| 87 | *Haemaphysalis longicornis* | Jilin-Dunhua | Negative | 13 |
| 88 | *Ixodes persulcatus* | Jilin-Jilin | Negative | 11 |
| 89 | *Ixodes persulcatus* | Jilin-Jilin | Negative | 13 |
| 90 | *Dermacentor nuttalli* | Jilin-Jilin | *Rickettsia raoultii* | 6 |
| 91 | *Haemaphysalis longicornis* | Jilin-Jilin | Negative | 18 |
| 92 | *Haemaphysalis longicornis* | Jilin-Dunhua | Negative | 28 |
| 93 | *Ixodes persulcatus* | Jilin-Dunhua | Negative | 14 |
| 94 | *Haemaphysalis longicornis* | Jilin-Dunhua | Negative | 13 |
| 95 | *Dermacentor nuttalli* | Jilin-Dunhua | *Rickettsia raoultii* | 13 |
| 96 | *Ixodes persulcatus* | Heilongjiang-Jiamusi | Negative | 14 |
| 97 | *Ixodes persulcatus* | Heilongjiang-Jiamusi | Negative | 15 |
| 98 | *Ixodes persulcatus* | Heilongjiang-Jiamusi | "*Candidatus* Rickettsia tarasevichiae" | 15 |
| 99 | *Ixodes persulcatus* | Heilongjiang-Jiamusi | Negative | 15 |
| 100 | *Ixodes persulcatus* | Heilongjiang-Jiamusi | Negative | 15 |
| 101 | *Ixodes persulcatus* | Heilongjiang-Jiamusi | "*Candidatus* Rickettsia tarasevichiae" | 15 |
| 102 | *Ixodes persulcatus* | Heilongjiang-Jiamusi | "*Candidatus* Rickettsia tarasevichiae" | 15 |
| 103 | *Ixodes persulcatus* | Heilongjiang-Jiamusi | "*Candidatus* Rickettsia tarasevichiae" | 15 |
| 104 | *Ixodes persulcatus* | Heilongjiang-Jiamusi | "*Candidatus* Rickettsia tarasevichiae" | 15 |
| 105 | *Ixodes persulcatus* | Heilongjiang-Jiamusi | Negative | 15 |
| 106 | *Ixodes persulcatus* | Heilongjiang-Jiamusi | "*Candidatus* Rickettsia tarasevichiae" | 15 |
| 107 | *Ixodes persulcatus* | Heilongjiang-Jiamusi | "*Candidatus* Rickettsia tarasevichiae" | 15 |
| 108 | *Ixodes persulcatus* | Heilongjiang-Jiamusi | Negative | 15 |
| 109 | *Ixodes persulcatus* | Heilongjiang-Jiamusi | Negative | 15 |
| 110 | *Ixodes persulcatus* | Heilongjiang-Jiamusi | "*Candidatus* Rickettsia tarasevichiae" | 15 |
| 111 | *Ixodes persulcatus* | Heilongjiang-Jiamusi | "*Candidatus* Rickettsia tarasevichiae" | 15 |
| 112 | *Ixodes persulcatus* | Heilongjiang-Jiamusi | "*Candidatus* Rickettsia tarasevichiae" | 15 |
| 113 | *Ixodes persulcatus* | Heilongjiang-Jiamusi | "*Candidatus* Rickettsia tarasevichiae" | 15 |
| 114 | *Ixodes persulcatus* | Heilongjiang-Jiamusi | "*Candidatus* Rickettsia tarasevichiae" | 14 |
| 115 | *Ixodes persulcatus* | Heilongjiang-Jiamusi | Negative | 14 |
| 116 | *Ixodes persulcatus* | Heilongjiang-Jiamusi | "*Candidatus* Rickettsia tarasevichiae" | 14 |
| 117 | *Ixodes persulcatus* | Heilongjiang-Jiamusi | Negative | 14 |
| 118 | *Ixodes persulcatus* | Heilongjiang-Jiamusi | "*Candidatus* Rickettsia tarasevichiae" | 15 |
| 119 | *Ixodes persulcatus* | Heilongjiang-Jiamusi | "*Candidatus* Rickettsia tarasevichiae" | 15 |
| 120 | *Ixodes persulcatus* | Heilongjiang-Jiamusi | Negative | 15 |
| 121 | *Ixodes persulcatus* | Heilongjiang-Jiamusi | Negative | 15 |
| 122 | *Ixodes persulcatus* | Heilongjiang-Jiamusi | "*Candidatus* Rickettsia tarasevichiae" | 15 |
| 123 | *Ixodes persulcatus* | Heilongjiang-Jiamusi | "*Candidatus* Rickettsia tarasevichiae" | 15 |
| 124 | *Ixodes persulcatus* | Heilongjiang-Jiamusi | Negative | 15 |
| 125 | *Ixodes persulcatus* | Heilongjiang-Jiamusi | Negative | 15 |
| 126 | *Ixodes persulcatus* | Heilongjiang-Jiamusi | "*Candidatus* Rickettsia tarasevichiae" | 15 |
| 127 | *Ixodes persulcatus* | Heilongjiang-Jiamusi | Negative | 15 |
| 128 | *Ixodes persulcatus* | Heilongjiang-Jiamusi | Negative | 15 |
| 129 | *Ixodes persulcatus* | Heilongjiang-Jiamusi | "*Candidatus* Rickettsia tarasevichiae" | 15 |
| 130 | *Haemaphysalis concinna* | Heilongjiang-Shuangyashan | Negative | 15 |
| 131 | *Haemaphysalis concinna* | Heilongjiang-Shuangyashan | *Rickettsia heilongjiangensis* | 15 |
| 132 | *Haemaphysalis concinna* | Heilongjiang-Shuangyashan | "*Candidatus* Rickettsia tarasevichiae" | 15 |
| 134 | *Haemaphysalis concinna* | Heilongjiang-Shuangyashan | Negative | 15 |
| 135 | *Ixodes persulcatus* | Heilongjiang-Shuangyashan | "*Candidatus* Rickettsia tarasevichiae" | 15 |
| 136 | *Ixodes persulcatus* | Heilongjiang-Shuangyashan | Negative | 15 |
| 137 | *Ixodes persulcatus* | Heilongjiang-Shuangyashan | "*Candidatus* Rickettsia tarasevichiae" | 15 |
| 138 | *Ixodes persulcatus* | Heilongjiang-Shuangyashan | Negative | 14 |
| 139 | *Ixodes persulcatus* | Heilongjiang-Shuangyashan | Negative | 14 |
| 142 | *Ixodes persulcatus* | Heilongjiang-Shuangyashan | "*Candidatus* Rickettsia tarasevichiae" | 14 |
| 143 | *Ixodes persulcatus* | Heilongjiang-Shuangyashan | "*Candidatus* Rickettsia tarasevichiae" | 14 |
| 144 | *Ixodes persulcatus* | Heilongjiang-Shuangyashan | Negative | 14 |
| 145 | *Ixodes persulcatus* | Heilongjiang-Shuangyashan | "*Candidatus* Rickettsia tarasevichiae" | 14 |
| 146 | *Ixodes persulcatus* | Heilongjiang-Shuangyashan | "*Candidatus* Rickettsia tarasevichiae" | 14 |
| 147 | *Ixodes persulcatus* | Heilongjiang-Shuangyashan | Negative | 16 |
| 148 | *Ixodes persulcatus* | Heilongjiang-Shuangyashan | "*Candidatus* Rickettsia tarasevichiae" | 16 |
| 149 | *Ixodes persulcatus* | Heilongjiang-Shuangyashan | "*Candidatus* Rickettsia tarasevichiae" | 16 |
| 150 | *Ixodes persulcatus* | Heilongjiang-Shuangyashan | "*Candidatus* Rickettsia tarasevichiae" | 15 |
| 151 | *Ixodes persulcatus* | Heilongjiang-Shuangyashan | "*Candidatus* Rickettsia tarasevichiae" | 15 |
| 152 | *Ixodes persulcatus* | Heilongjiang-Shuangyashan | "*Candidatus* Rickettsia tarasevichiae" | 15 |
| 153 | *Ixodes persulcatus* | Heilongjiang-Shuangyashan | "*Candidatus* Rickettsia tarasevichiae" | 15 |
| 154 | *Ixodes persulcatus* | Heilongjiang-Shuangyashan | "*Candidatus* Rickettsia tarasevichiae" | 15 |
| 155 | *Ixodes persulcatus* | Heilongjiang-Shuangyashan | "*Candidatus* Rickettsia tarasevichiae" | 15 |
| 156 | *Ixodes persulcatus* | Heilongjiang-Shuangyashan | Negative | 15 |
| 157 | *Ixodes persulcatus* | Heilongjiang-Shuangyashan | "*Candidatus* Rickettsia tarasevichiae" | 15 |
| 158 | *Ixodes persulcatus* | Heilongjiang-Shuangyashan | Negative | 15 |
| 159 | *Ixodes persulcatus* | Heilongjiang-Shuangyashan | Negative | 15 |
| 160 | *Ixodes persulcatus* | Heilongjiang-Shuangyashan | "*Candidatus* Rickettsia tarasevichiae" | 15 |
| 161 | *Haemaphysalis concinna* | Heilongjiang-Shuangyashan | Negative | 12 |
| 164 | *Dermacentor silvarum* | Heilongjiang-Jixi | *Rickettsia raoultii* | 13 |
| 165 | *Dermacentor nuttalli* | Heilongjiang-Jixi | *Rickettsia raoultii* | 12 |
| 166 | *Dermacentor nuttalli* | Heilongjiang-Jixi | *Rickettsia raoultii* | 12 |
| 168 | *Ixodes persulcatus* | Heilongjiang-Suifenhe | "*Candidatus* Rickettsia tarasevichiae" | 14 |
| 169 | *Ixodes persulcatus* | Heilongjiang-Suifenhe | Negative | 14 |
| 170 | *Ixodes persulcatus* | Heilongjiang-Suifenhe | "*Candidatus* Rickettsia tarasevichiae" | 13 |
| 173 | *Haemaphysalis concinna* | Heilongjiang-Suifenhe | *Rickettsia heilongjiangensis* | 14 |
| 174 | *Haemaphysalis concinna* | Heilongjiang-Suifenhe | *Rickettsia heilongjiangensis* | 14 |
| 175 | *Haemaphysalis concinna* | Heilongjiang-Suifenhe | Negative | 14 |
| 176 | *Haemaphysalis concinna* | Heilongjiang-Suifenhe | *Rickettsia heilongjiangensis* | 13 |
| 178 | *Haemaphysalis longicornis* | Heilongjiang-Suifenhe | *Rickettsia heilongjiangensis* | 14 |
| 179 | *Ixodes persulcatus* | Heilongjiang-Suifenhe | "*Candidatus* Rickettsia tarasevichiae" | 17 |
| 180 | *Ixodes persulcatus* | Heilongjiang-Suifenhe | "*Candidatus* Rickettsia tarasevichiae" | 17 |
| 181 | *Ixodes persulcatus* | Heilongjiang-Suifenhe | "*Candidatus* Rickettsia tarasevichiae" | 16 |
| 182 | *Dermacentor silvarum* | Heilongjiang-Tongjiang | *Rickettsia raoultii* | 14 |
| 183 | *Dermacentor nuttalli* | Heilongjiang-Tongjiang | *Rickettsia raoultii* | 10 |
| 184 | *Dermacentor nuttalli* | Heilongjiang-Tongjiang | *Rickettsia raoultii* | 10 |
| 185 | *Ixodes persulcatus* | Heilongjiang-Tongjiang | "*Candidatus* Rickettsia tarasevichiae" | 9 |
| 186 | *Haemaphysalis concinna* | Heilongjiang-Tongjiang | Negative | 17 |
| 187 | *Haemaphysalis concinna* | Heilongjiang-Tongjiang | *Rickettsia heilongjiangensis* | 17 |
| 188 | *Haemaphysalis longicornis* | Heilongjiang-Tongjiang | *Rickettsia heilongjiangensis* | 11 |
| 189 | *Haemaphysalis longicornis* | Heilongjiang-Tongjiang | Negative | 10 |
| 190 | *Ixodes persulcatus* | Heilongjiang-Yichun | Negative | 15 |
| 191 | *Ixodes persulcatus* | Heilongjiang-Yichun | "*Candidatus* Rickettsia tarasevichiae" | 15 |
| 192 | *Ixodes persulcatus* | Heilongjiang-Yichun | Negative | 15 |
| 193 | *Ixodes persulcatus* | Heilongjiang-Yichun | "*Candidatus* Rickettsia tarasevichiae" | 15 |
| 194 | *Ixodes persulcatus* | Heilongjiang-Yichun | "*Candidatus* Rickettsia tarasevichiae" | 15 |
| 195 | *Ixodes persulcatus* | Heilongjiang-Yichun | "*Candidatus* Rickettsia tarasevichiae" | 15 |
| 196 | *Ixodes persulcatus* | Heilongjiang-Yichun | "*Candidatus* Rickettsia tarasevichiae" | 15 |
| 197 | *Ixodes persulcatus* | Heilongjiang-Yichun | "*Candidatus* Rickettsia tarasevichiae" | 15 |
| 198 | *Ixodes persulcatus* | Heilongjiang-Yichun | "*Candidatus* Rickettsia tarasevichiae" | 15 |
| 199 | *Ixodes persulcatus* | Heilongjiang-Yichun | "*Candidatus* Rickettsia tarasevichiae" | 15 |
| 200 | *Ixodes persulcatus* | Heilongjiang-Yichun | "*Candidatus* Rickettsia tarasevichiae" | 15 |
| 201 | *Haemaphysalis concinna* | Heilongjiang-Yichun | *Rickettsia heilongjiangensis* | 14 |
| 202 | *Haemaphysalis concinna* | Heilongjiang-Yichun | *Rickettsia heilongjiangensis* | 14 |
| 203 | *Haemaphysalis concinna* | Heilongjiang-Yichun | Negative | 14 |
| 204 | *Haemaphysalis concinna* | Heilongjiang-Yichun | Negative | 14 |
| 205 | *Haemaphysalis concinna* | Heilongjiang-Yichun | *Rickettsia heilongjiangensis* | 15 |
| 206 | *Haemaphysalis concinna* | Heilongjiang-Yichun | Negative | 15 |
| 208 | *Haemaphysalis concinna* | Heilongjiang-Yichun | Negative | 15 |
| 209 | *Haemaphysalis concinna* | Heilongjiang-Yichun | *Rickettsia heilongjiangensis* | 15 |
| 210 | *Haemaphysalis concinna* | Heilongjiang-Yichun | *Rickettsia heilongjiangensis* | 15 |
| 211 | *Haemaphysalis concinna* | Heilongjiang-Yichun | Negative | 15 |
| 212 | *Haemaphysalis concinna* | Heilongjiang-Yichun | *Rickettsia heilongjiangensis* | 15 |
| 214 | *Haemaphysalis concinna* | Heilongjiang-Yichun | *Rickettsia heilongjiangensis* | 15 |
| 215 | *Haemaphysalis concinna* | Heilongjiang-Yichun | Negative | 15 |
| 216 | *Haemaphysalis concinna* | Heilongjiang-Yichun | *Rickettsia heilongjiangensis* | 15 |
| 217 | *Haemaphysalis concinna* | Heilongjiang-Yichun | *Rickettsia heilongjiangensis* | 15 |
| 218 | *Haemaphysalis concinna* | Heilongjiang-Yichun | *Rickettsia heilongjiangensis* | 15 |
| 219 | *Haemaphysalis concinna* | Heilongjiang-Yichun | Negative | 15 |
| 220 | *Haemaphysalis longicornis* | Heilongjiang-Yichun | *Rickettsia heilongjiangensis* | 13 |
| 222 | *Haemaphysalis longicornis* | Heilongjiang-Yichun | Negative | 14 |
| 223 | *Haemaphysalis longicornis* | Heilongjiang-Yichun | Negative | 14 |
| 224 | *Haemaphysalis longicornis* | Heilongjiang-Yichun | *Rickettsia heilongjiangensis* | 14 |
| 225 | *Haemaphysalis longicornis* | Heilongjiang-Yichun | Negative | 14 |
| 226 | *Haemaphysalis longicornis* | Heilongjiang-Yichun | *Rickettsia heilongjiangensis* | 14 |
| 227 | *Haemaphysalis longicornis* | Heilongjiang-Yichun | Negative | 14 |
| 228 | *Haemaphysalis longicornis* | Heilongjiang-Yichun | *Rickettsia heilongjiangensis* | 14 |
| 229 | *Ixodes persulcatus* | Heilongjiang-Yichun | "*Candidatus* Rickettsia tarasevichiae" | 15 |
| 230 | *Ixodes persulcatus* | Heilongjiang-Yichun | "*Candidatus* Rickettsia tarasevichiae" | 15 |
| 232 | *Ixodes persulcatus* | Heilongjiang-Yichun | "*Candidatus* Rickettsia tarasevichiae" | 15 |
| 233 | *Ixodes persulcatus* | Heilongjiang-Yichun | "*Candidatus* Rickettsia tarasevichiae" | 14 |
| 234 | *Ixodes persulcatus* | Heilongjiang-Yichun | "*Candidatus* Rickettsia tarasevichiae" | 14 |
| 235 | *Ixodes persulcatus* | Heilongjiang-Yichun | "*Candidatus* Rickettsia tarasevichiae" | 14 |
| 236 | *Ixodes persulcatus* | Heilongjiang-Yichun | "*Candidatus* Rickettsia tarasevichiae" | 14 |
| 237 | *Ixodes persulcatus* | Heilongjiang-Yichun | "*Candidatus* Rickettsia tarasevichiae" | 14 |
| 238 | *Ixodes persulcatus* | Heilongjiang-Yichun | "*Candidatus* Rickettsia tarasevichiae" | 14 |
| 239 | *Ixodes persulcatus* | Heilongjiang-Yichun | "*Candidatus* Rickettsia tarasevichiae" | 14 |
| 241 | *Ixodes persulcatus* | Heilongjiang-Jixi | "*Candidatus* Rickettsia tarasevichiae" | 4 |
| 242 | *Dermacentor nuttalli* | Heilongjiang-Suifenhe | *Rickettsia raoultii* | 3 |
| 243 | *Ixodes persulcatus* | Heilongjiang-Tongjiang | "*Candidatus* Rickettsia tarasevichiae" | 3 |
| 244 | *Dermacentor silvarum* | Heilongjiang-Yichun | *Rickettsia raoultii* | 2 |
| **Total** |  |  |  | 2,928 |

*The sample ID is discrete. There is a total of 204 pools
