# Supplementary figures and images for: Characterization of rickettsiae in ticks in northeastern China
Source: Parasit Vectors. 2016 Sep 13;9(1):498. doi: 10.1186/s13071-016-1764-2 (PMC5022169; doi:10.1186/s13071-016-1764-2)

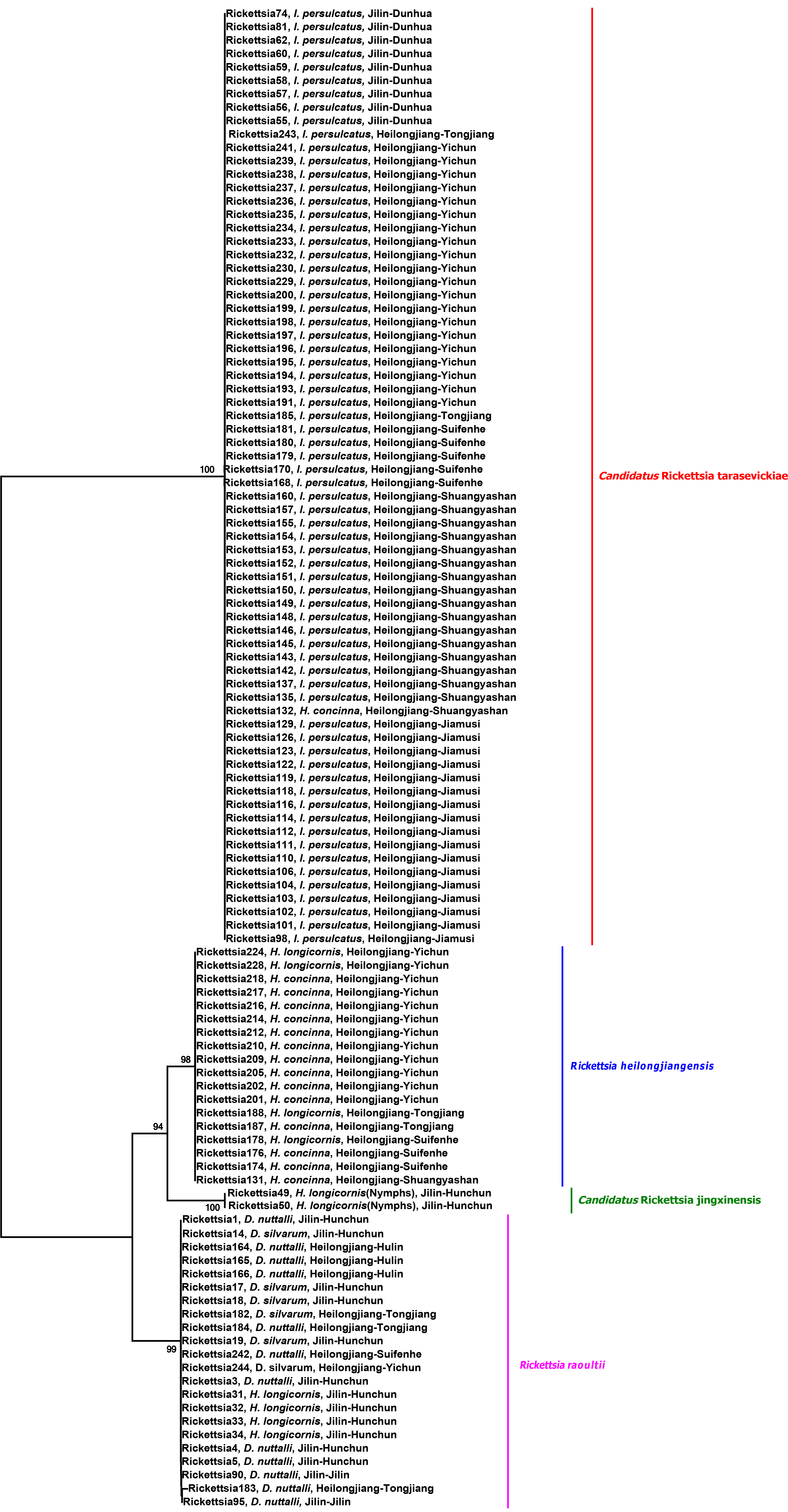

Supplement: Additional file 2: — Figure S1. Phylogenetic analysis of the partial ompA (533 bp) sequences obtained in this study. Targeted fragments of ompA gene of all of the positive samples were analyzed through the Maximum Likelihood method using MEGA 6.0 [40]. Phylogenic tree was tested by bootstrapping (1000 pseudoreplicates). Sequences in the tree are identified by sample number, tick species, and sampling site. (TIF 5444 kb) [file 13071_2016_1764_MOESM2_ESM.tif]
